# Massively parallel sequencing of the mouse exome to accurately identify rare, induced mutations: an immediate source for thousands of new mouse models

**DOI:** 10.1098/rsob.120061

**Published:** 2012-05

**Authors:** T. D. Andrews, B. Whittle, M. A. Field, B. Balakishnan, Y. Zhang, Y. Shao, V. Cho, M. Kirk, M. Singh, Y. Xia, J. Hager, S. Winslade, G. Sjollema, B. Beutler, A. Enders, C. C. Goodnow

**Affiliations:** 1 Immunogenomics Laboratory, Australian National University, GPO Box 334, Canberra City, Australian Capital Territory, 2601, Australia; 2 Ramaciotti Immunisation Genomics Laboratory, John Curtin School of Medical Research, Australian National University, GPO Box 334, Canberra City, Australian Capital Territory, 2601, Australia; 3 Australian Phenomics Facility, Australian National University, Hugh Ennor Building, Building 117, Garran Road, Canberra City, Australian Capital Territory, 0200, Australia; 4 Center for the Genetics of Host Defense, University of Texas Southwestern, 6000 Harry Hines Boulevard, Dallas, TX 75930-8505, USA; 5 Department of Genetics, Scripps Research Institute, 10550 North Torrey Pines Road, La Jolla, CA 92037, USA; 6 Centre National de Génotypage, 2 rue Gaston Crémieux, CP5721, 91057, Evry Cedex, France

**Keywords:** exome sequencing, DNA capture, *N*-ethyl-*N*-nitrosourea mutagenesis, variation detection, mutation detection, mouse

## Abstract

Accurate identification of sparse heterozygous single-nucleotide variants (SNVs) is a critical challenge for identifying the causative mutations in mouse genetic screens, human genetic diseases and cancer. When seeking to identify causal DNA variants that occur at such low rates, they are overwhelmed by false-positive calls that arise from a range of technical and biological sources. We describe a strategy using whole-exome capture, massively parallel DNA sequencing and computational analysis, which identifies with a low false-positive rate the majority of heterozygous and homozygous SNVs arising de novo with a frequency of one nucleotide substitution per megabase in progeny of *N*-ethyl-*N*-nitrosourea (ENU)-mutated C57BL/6j mice. We found that by applying a strategy of filtering raw SNV calls against known and platform-specific variants we could call true SNVs with a false-positive rate of 19.4 per cent and an estimated false-negative rate of 21.3 per cent. These error rates are small enough to enable calling a causative mutation from both homozygous and heterozygous candidate mutation lists with little or no further experimental validation. The efficacy of this approach is demonstrated by identifying the causative mutation in the *Ptprc* gene in a lymphocyte-deficient strain and in 11 other strains with immune disorders or obesity, without the need for meiotic mapping. Exome sequencing of first-generation mutant mice revealed hundreds of unphenotyped protein-changing mutations, 52 per cent of which are predicted to be deleterious, which now become available for breeding and experimental analysis. We show that exome sequencing data alone are sufficient to identify induced mutations. This approach transforms genetic screens in mice, establishes a general strategy for analysing rare DNA variants and opens up a large new source for experimental models of human disease.

## 2. Introduction

Genetic traits in mammals have long posed a great challenge in connecting them to their causal DNA variant. This is especially true when that variant is a single-nucleotide substitution and is present on only one of the two copies of a chromosome. Finding such a single-nucleotide substitution in a genome as large as humans or mice without huge numbers of false positives and without reducing the search to a sub-chromosomal region by meiotic mapping has been an unattainable goal. Single-nucleotide variants (SNVs) represent a major source of de novo and inherited genomic variation in humans, mice and other mammals, and, as such, new strategies are needed to identify and analyse these variants accurately on a genome-wide scale.

Genetic analyses of mammalian traits are often performed in inbred C57BL/6 laboratory mice. These mice have a known homogeneous reference genome sequence and have a uniform genetic background that allows experimental reproducibility and transplantation experiments. In these mice, treatment with the chemical mutagen *N*-ethyl-*N*-nitrosourea (ENU) efficiently generates random single-base mutations in the germline DNA (reviewed in [1]). Diseases and traits resulting from these ENU-induced mutations can be detected by phenotypic screening procedures relevant to an area of biological investigation.

The bottleneck of the ENU mutagenesis approach has long been in identifying a single disease-causing mutation in an entire genome of possibilities. Until recently, the approach employed has been arduous: to out-cross affected mice to another inbred strain and then use a panel of common strain-specific variants to meiotically map the causal mutation to a sub-region of an individual chromosome of less than 20 megabases (Mb). Once limited to a relatively short list of positional candidate genes, PCR amplification of all exons in the mapped interval followed by Sanger sequencing could then be performed and variants identified by a combination of automated and manual review of the sequence traces. This has proven to be an effective strategy, although it can take several years and is labour-intensive, expensive and often confounded by modifier genes introduced during the cross to another inbred strain.

To date, all but the smallest minority of causative ENU-induced mutations have been shown to reside in the exonic portion of the genome. Approximately 75 per cent are caused by SNVs in protein-coding exons that result in missense or nonsense mutations and the remaining approximately 25 per cent are SNVs in splice donor–acceptor sites that disrupt correct mRNA splicing to cause protein truncations, deletions or nonsense-mediated decay [2]. Hence, sequencing of the exome rather than the whole genome should identify almost all interesting ENU-induced variants. Array- and solution-based DNA capture technologies [3,4] can now reliably enrich a DNA sample for coding regions, enabling massively parallel sequencing to be undertaken on a greatly reduced proportion of the genome. Exome capture followed by sequencing has already become an established technique in human genetics and an early vanguard of reports has identified the genetic cause of a number of monogenic diseases (reviewed in [5]). In most of these studies, prior information regarding a general chromosomal location of the genetic lesion was known, heritability information was available or a candidate gene approach was used. One feature of all of these studies was the difficulty in discerning causative, deleterious mutations from normal genetic variation and sequencing errors.

In the mouse, early studies [6–8] using slightly different approaches have identified ENU-induced mutations using massively parallel sequencing information. Zhang *et al.* [8] identified a previously known ENU-induced mutant by sequencing cloned bacterial artificial chromosomes from a 2.2 Mb genomic region that had first been defined by meiotic mapping. Arnold *et al.* [6] applied shallow sequencing of the entire mouse genome to detect putative mutations and, following this, they performed extensive validation by Sanger sequencing and meiotic mapping. Yabas *et al.* [7] mapped a novel ENU mutation to a region of the X-chromosome, and identified the mutation by oligonucleotide bait-mediated capture and deep sequencing of exonic DNA fragments within this region. Fairfield *et al.* [9] provided an extensive demonstration of the utility of exome capture technology for identifying both homozygous and heterozygous ENU-induced and spontaneous mutations in nine mouse strains. However, in all cases these studies relied on at least coarse meiotic mapping information or considerable validation of SNV calls to identify the causative mutation. Fairfield *et al.* [9] suggest that an exome sequence as a sole source of information may not be enough to identify disease-causing induced mutations without extensive SNV validation.

In this study, we have investigated whether exome capture followed by sequencing provides sufficient information alone to reliably identify the rare, ENU-induced, de novo mutations in C57BL/6j mice. We generated exome datasets for 12 mutant mouse strains, including a matched technical and biological replicate dataset for one strain. We present methodology developed to identify both homozygous and heterozygous ENU-induced mutations and use this to identify 12 primary causative mutations and two disease-causing incidental mutations. We also reveal hundreds of potentially deleterious ENU mutations in first-generation (G1) mice that are immediately available for phenotypic and experimental analysis in their progeny. Our results demonstrate that exome sequencing provides highly reliable information which by itself is sufficient to identify ENU-induced mutations selected either by phenotype or by the nature of the gene that is mutated. These results provide an immediate source for thousands of new experimental models for understanding human diseases and establish a strategy that can be extended for identifying rare SNVs in outbred mice, humans and other species.

## 3. Results

### 3.1. Generation and detection of induced, de novo single-nucleotide variants

**Figure 1.**
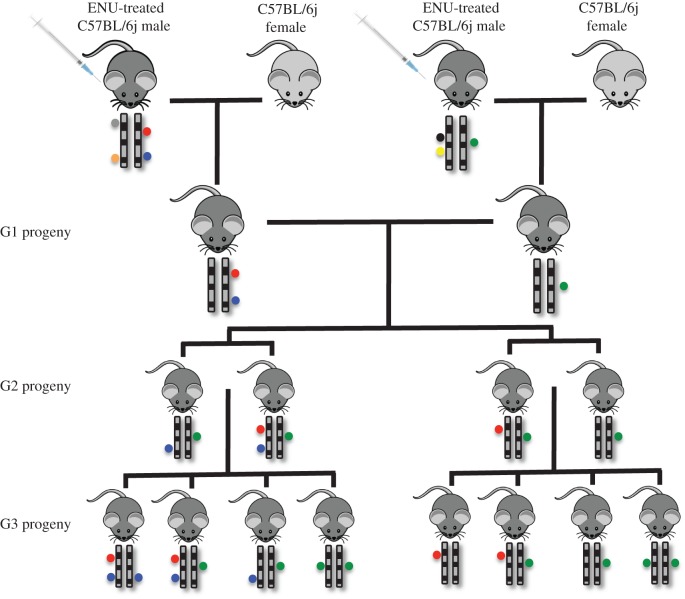
Summary of the structure of ENU-mutated mouse pedigrees. Each pedigree is initiated by two unrelated G1 founders. Each of these founders inherits a random set of de novo point mutations (coloured circles) on the paternal chromosomes, induced by ENU treatment of their male parent. These G1 founders will carry on average one to two DNA variants per Mb and 90 exonic ENU-induced mutations. Second-generation (G2) progeny of these mice inherit a theoretical 45 ENU-induced exonic mutations, all of which are carried in the heterozygous state. Two productive sibling–sibling matings of the G2 mice result in third-generation (G3) progeny that carry approximately 94% of the founding ENU-induced, protein-coding mutations, of which on average five are homozygous in any given mouse.

Many parallel mouse pedigrees, each segregating a different set of random, de novo mutations induced in the C57BL/6j genome by ENU were established using the breeding strategy shown in figure 1. Each pedigree was founded by two unrelated G1 mice conceived from male C57BL/6j mice that had been treated with three doses of ENU administered at 90 mg kg^−1^ to induce random point mutations in spermatogonial stem cells [2,10,11]. Based on published mutation rates [12–14], we estimated that each of these G1 animals would carry approximately one de novo SNV per Mb of the paternal genome, of which around 45 would result in a non-synonymous exonic mutation. Intercrossing of the G1 animals transmitted half of these mutations in heterozygous state to each of their second-generation (G2) offspring. Intercrossing the G2 animals subsequently transmitted approximately 94 per cent of the mutations to offspring, a subset of which was inherited in homozygous state in third-generation (G3) animals (figure 1).

**Figure 2.**
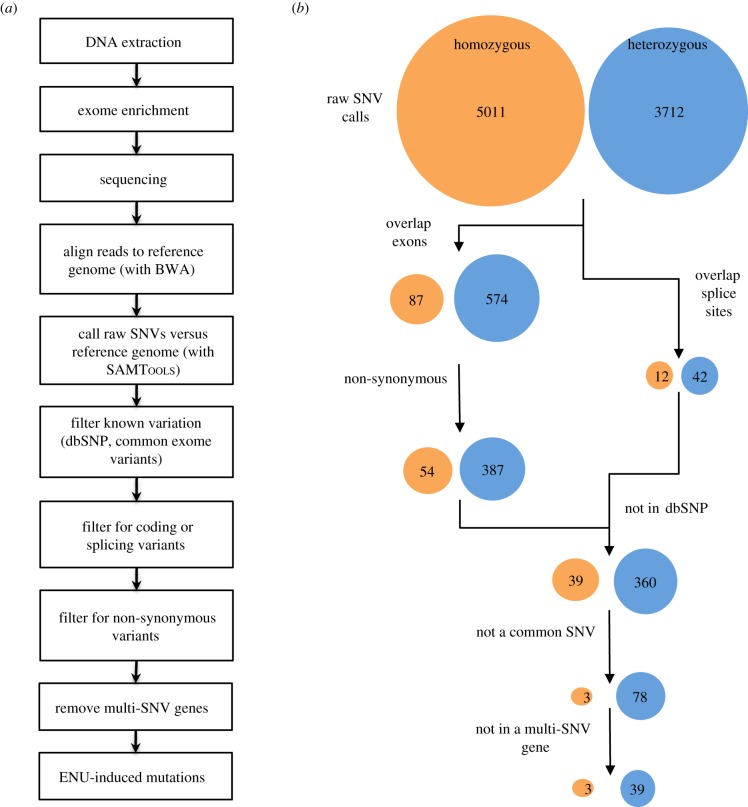
Workflow and filtering strategy used to identify de novo protein-changing mutations. (*a*) Following DNA extraction, exome enrichment and sequencing, reads were aligned to the mouse reference genome [15] using BWA [16] and variation between the two genomes identified using SAMTools [17]. The set of raw SNVs was subsequently filtered to annotate known variation and other apparent SNVs known not to be ENU-induced. SNVs were further filtered to annotate those that fell within coding regions (or adjacent splice donor/acceptor sites) and were non-synonymous changes. Finally, as ENU treatment is known to introduce a uniform genomic distribution of mutations, genes that contained multiple SNVs were filtered from the final set of variants. (*b*) Using this cumulative filtering strategy against a single replicate exome sequence of the *nimbus* mouse, the initial 8723 variant calls reduced to a final set of three homozygous and 39 heterozygous putative mutations. Circles representing homozygous and heterozygous SNV numbers are coloured orange and blue, respectively.

We developed a workflow (figure 2*a*) to use massively parallel sequencing reads as a sole data source to identify exonic ENU-induced mutations in 15 DNA samples taken from mutated mice (see electronic supplementary material, table S1). These samples were prepared and enriched for exonic sequences using either Agilent or Nimblegen solution-based capture technologies. Each exome sample was then sequenced as paired-end reads in a full lane of an Illumina GAIIx sequencer or as a multiplexed, bar-coded sample in an Illumina HiSeq sequencer, and the resultant reads aligned to the C57BL/6 mouse reference genome using the BWA aligner [16]. Table S1 in the electronic supplementary material shows the numbers of reads sequenced and the number of reads aligned to exonic target regions per sample. The exome capture efficiency was uniformly high with approximately 40 to 55 per cent of all DNA sequenced being exonic. Based on a mouse genome size of 2493 Mb [15] and 37 Mb of exonic sequence, using consensus coding sequence (CCDS) exons [18], this represents on average a 30.6-fold (*σ* = 3.3) sequence enrichment. Across the coding portion of the genome sequence, coverage was generally better than 85 per cent at 5 times depth and better than 70 per cent at 20 times depth, although coverage was distinctly less for the sex chromosomes (see electronic supplementary material, figure S1).

Raw SNVs relative to the C57BL/6 reference sequence were called using SAMTools [17]. In the inbred C57BL/6j mice we analysed, we would expect the number of true variant calls to be low (approx. 50 exonic SNVs) and almost entirely due to ENU treatment of the G0 male mouse that founded their line. However, in each animal, of the order of 10 000 raw SNVs were called across the entire genome, of which 500–750 SNV calls were located in exons and/or near exon splice sites (see electronic supplementary material, table S2). Multiple sources can be attributed to these variant calls, potentially being due to genetic drift of the C57BL/6j mouse strain versus the reference genome and the frequency of sequencing errors in massively parallel sequencing. However, many of the variants appear to be called because of technical issues associated with aligning large numbers of short reads to a large genome containing repeated or highly similar sequence regions. To reduce these raw variant calls to a smaller number highly enriched for ENU-induced mutations, we applied a series of filters to remove known variants (present in dbSNP) and/or recurrent false-positive variants (figure 2*a*). We assert that between multiple, unrelated mouse exome sequences, de novo ENU-induced nucleotide changes should be unique to individual pedigrees, whereas other sources of variants should recur. Based on this reasoning, we collated a list of SNVs that recurred in more than one unrelated mouse and found this list to be a very effective filter for false-positive and potentially sequencer- and enrichment-specific variants. A further filter was applied to remove variants where they originate from a gene with multiple SNV calls, assuming that in any single ENU-mutated mouse it is highly unlikely that the same gene will have multiple mutations and that the calls are due to incorrect alignment of sequence reads between members of gene families. Figure 2*b* shows the efficacy of each individual filtering step applied and the outcome of the filters applied in a cumulative manner. Overall, from a set of several thousands of raw variant calls, the cumulative filtering reduced this number mostly to less than 10 homozygous and 50 heterozygous exonic variants per mouse (see electronic supplementary material, table S2), closely approximating the expected rate (figure 1).

### 3.2. Sensitivity and specificity of single-nucleotide variant detection

**Figure 3.**
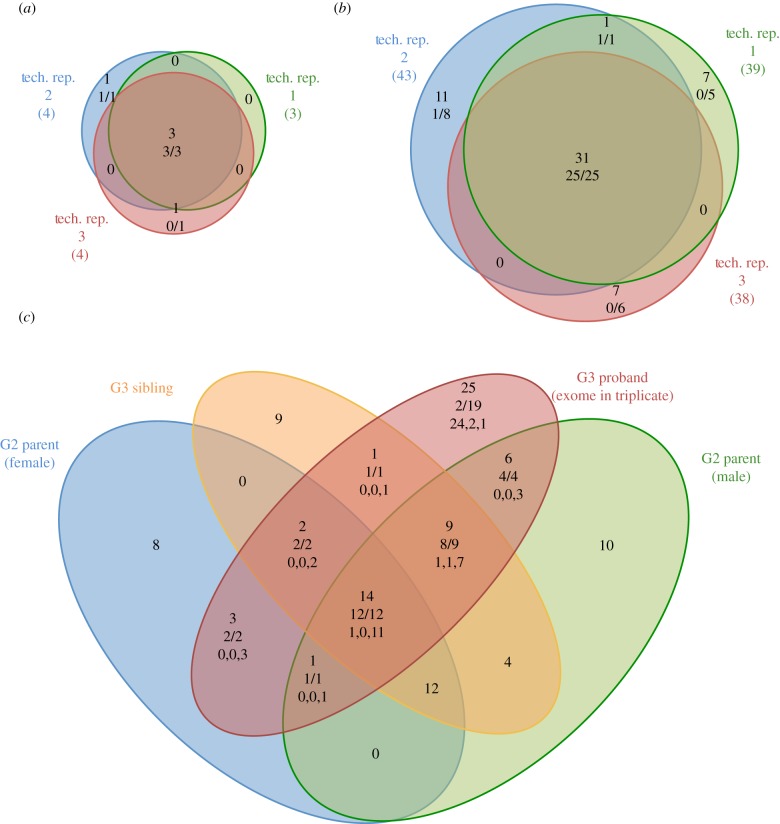
Sensitivity and specificity of mutation detection in the *nimbus* mutant mouse pedigree assessed through technical and biological replicate datasets. Venn diagrams of overlap of filtered variant calls between three technical replicate exome sequence datasets, showing putative (*a*) homozygous and (*b*) heterozygous ENU-induced mutations. The red, green and blue circles each indicate separate technical replicates, and the coloured numbers associated with each denote the total number of variants called in each dataset. Upper numbers within each sector show the number of filter-passing SNVs called in one, two or all three technical replicates. The numbers below show the fraction of these SNVs that were validated as true mutations by independent, custom, SNV-specific PCR assays. The denominator in each case is the number of SNVs where an SNV-specific PCR assay was established successfully. (*c*) Overlap of filtered variant calls from a set of four biological replicates, representing two parental G2 *nimbus* mice and two of their G3 offspring. One of the G3 offspring (labelled G3 proband) is the same mouse as that sequenced in the technical replicates shown in (*a*) and (*b*). The variant numbers shown for this mouse are pooled values from the three technical replicates. Both G2 *nimbus* mice and the sibling of the G3 proband (labelled G3 sibling) are unaffected by the lymphopaenia phenotype. Upper numbers within each sector of the four-way Venn diagram show the total number of filter-passing heterozygous and homozygous SNVs called in one or more of the replicates from this pedigree. The numbers immediately below show the fractions of biologically replicated SNVs that were validated as true mutations by independent, custom, SNV-specific PCR assays. In the case of technically replicated data from the proband (the red circle), the third line of data in each region of overlap shows the number of times a variant was seen in one, two or three replicates (formatted as: single count, double count and triple count).

To assess the reliability of SNV calls made from a single exome dataset, we performed a technical and biological replication experiment on G2 and G3 animals from a pedigree (*nimbus*) that had shown mild lymphopaenia in the blood of some G3 offspring. These *nimbus* mutant animals displayed a fourfold reduction in the percentage of CD3^+^ T cells and represented 8 of a total of 30 phenotyped individuals, suggesting that *nimbus* was a recessive trait. We sequenced the exome of one proband G3 affected *nimbus* mouse in triplicate (technical replicates) and also sequenced the exome of both G2 parents and an unaffected G3 sibling (loosely termed biological replicates). Figure 3*a,b* shows that the SNVs called in each of the technical replicates of the proband's exome were highly replicable. The total number of coding changes called in each replicate was 47, 42 and 42, of which 34 were called in all three replicates, representing 72, 81 and 81 per cent of the SNVs called in each individual exome analysis. The triplicated SNV calls comprised three homozygous and 31 heterozygous mutations. We successfully established custom, SNV-specific PCR assays (Amplifluor assays; see §5.4) for 50 of the SNVs called in one or more of these replicates. From 50 successful assays, 100 per cent (28 of 28) of the triplicated SNV calls were validated as true mutations in this pedigree, whereas of the SNV calls that were present in only one or two of the replicate analyses only 14 per cent (3 of 22) were validated and the remainder were established to be false positives (figure 3*a,b* and table 1). From these technical replicate data the false-positive call rate among our filtered variants can be estimated as 19.4 per cent, calculated from an average of six false-positive calls per replicate exome as a proportion of the 31 true-positive SNVs.

**Table 1.** Validation results of all SNVs detected in proband replicate exome sequences. chr, chromosome; coord, coordinate; het, heterozygous; hom, homozygous; wt, wild type.

chr	coord	hom/het	wt genotype	genotype in G3 proband	genotype in G2 parent (female)	genotype in G2 parent (male)	genotype in G3 sibling	validated	number of replicates SNV detected	gene	amino acid change	PolyPhen2 score
1	6264360	het	C/C	C/T	C/C	C/T	C/C	yes	3	Rb1cc1	splice	—
1	59115542	het	A/A	A/T	A/T	A/T	T/T	yes	3	Als2cr11	S→T	0.126
1	139986182	hom	C/C	T/T	C/T	C/T	C/T	yes	3	Ptprc	splice	—
1	153543280	hom	T/T	A/A	T/A	T/A	T/A	yes	3	Fam129a	V→D	0.998
1	155590911	het	G/G	G/A	G/G	G/A	G/G	yes	3	Rgs16	D→N	0.812
2	13387686	hom/het	C/C	C/T	C/C	C/C	C/C	yes	2	Cubn	splice	—
2	14210637	het	A/A	A/T	A/A	A/T	A/T	yes	3	Mrc1	M→L	0.725
2	26263680	het	T/T	T/C	T/C	T/C	T/C	yes	3	Inpp5e	E→G	0.008
2	49590962	het	G/G	G/T	G/T	G/T	G/T	yes	3	Kif5c	V→L	0
2	70417236	het	A/A	A/G	A/G	A/G	A/G	yes	3	Gad1	T→A	0.512
2	89592814	het	A/A	A/T	A/T	A/A	A/A	yes	3	Olfr1253	M→K	0.988
3	19910826	het	C/C	C/A	C/C	C/A	C/A	yes	3	Hps3	V→L	0
3	88347367	het	A/A	A/G	A/A	A/G	A/G	yes	3	Rab25	V→A	0.098
3	108230315	het	A/A	A/T	A/A	A/A	A/T	yes	3	Sars	splice	—
4	15925782	het	A/A	A/T	A/T	A/A	A/T	yes	3	Osgin2	M→K	0.045
4	140271822	het	A/A	A/G	A/G	A/A	A/A	yes	3	Rcc2	I→V	0.178
6	34974302	het	A/A	A/G	A/G	A/G	G/G	yes	3	Cnot4	W→R	0.991
6	56952536	het	A/A	A/T	A/T	A/T	A/T	yes	1	Vmn1r6	D→V	0.028
6	116597609	het	A/A	A/T	A/T	T/T	A/A	yes	3	Rassf4	V→E	0.069
6	124882799	het	G/G	G/T	G/G	G/G	G/G	yes	3	Mlf2	G→V	0.999
9	108817748	het	T/T	T/C	T/T	T/C	T/T	yes	3	Tmem89	V→A	0.860
11	70159403	het	T/T	T/A	T/T	T/A	T/T	yes	3	Alox15	D→V	0.871
11	100051095	hom	T/T	C/C	T/C	T/C	T/C	yes	3	Krt9	K→E	0.144
11	120576524	het	T/T	T/A	T/A	T/T	T/A	yes	3	Lrrc45	S→T	0
13	34010014	het	T/T	T/A	T/T	T/A	T/A	yes	3	Serpinb6a	M→L	0
13	41141476	het	T/T	T/C	T/T	T/C	T/C	yes	3	Mak	N→S	0.001
14	99585815	het	A/A	A/G	A/G	A/G	G/G	yes	3	Pibf1	K→R	0.953
15	88956462	het	T/T	T/A	T/A	T/A	T/A	yes	3	Hdac10	Q→L	0.396
15	101398409	het	T/T	T/C	T/T	T/C	T/C	yes	3	Krt75	T→A	0.998
17	37362339	het	G/G	G/A	G/G	G/A	G/A	yes	3	Olfr96	E→K	0.001
17	37436474	het	T/T	T/C	T/T	T/C	T/C	yes	2	Olfr101	S→G	0
1	3661021	het	G/G	G/T	G/G	G/G	G/G	no	1	Xkr4		
1	26744177	het	A/A	A/G	A/A	A/G	A/G	no	1	4931408C20Rik		
4	43429551	het	A/A	A/C	A/A	A/A	A/A	no	1	Rusc2		
5	14934071	hom	G/G	C/C	G/G	G/G	G/G	no	1	RP23-239L21.1		
7	13629965	het	G/G	G/A	G/G	G/G	G/G	no	1	Mzf1		
7	66046516	het	C/C	C/A	C/C	C/C	C/C	no	1	Atp10a		
7	136751431	het	A/A	A/C	A/A	A/A	A/A	no	1	Wdr11		
9	40703661	het	C/C	C/A	C/C	C/C	C/C	no	1	4931429I11Rik		
10	36717792	het	C/C	C/A	C/C	C/C	C/C	no	1	Hdac2		
10	57861777	het	C/C	C/A	C/C	C/C	C/C	no	1	Lims1		
11	61265622	het	G/G	G/C	G/G	G/G	G/G	no	1	Rnf112		
11	69717525	het	C/C	C/A	C/C	C/C	C/C	no	1	Neurl4		
11	102527771	het	C/C	C/A	C/C	C/C	C/C	no	1	Gm1564		
12	21316065	het	C/C	C/G	C/C	C/C	C/C	no	1	Cpsf3		
14	65377451	het	G/G	G/T	G/G	G/G	G/G	no	1	Kif13b		
15	96846616	het	C/C	C/A	C/C	C/C	C/C	no	1	Slc38a4		
18	21288527	het	C/C	C/A	C/C	C/C	C/C	no	1	Fam59a		
X	121242115	het	A/A	A/G	A/A	A/A	A/A	no	1	Vmn2r121		
X	121246252	het	T/T	T/G	T/T	T/T	T/T	no	1	Vmn2r121		
1	56954912	het	A/A	A/G	A/G	A/G	G/G	no data	3	Satb2	C→R	0.537
1	74442070	het	A/A	A/C	A/A	A/A	A/A	no data	1	Ctdsp1	E→A	0.974
1	145475019	het	G/G	G/A	G/G	G/G	G/G	no data	1	Cdc73	splice	—
2	50148343	het	G/G	G/A	G/A	G/A	G/A	no data	3	Mmadhc	A→V	0
2	91831062	het	T/T	T/C	T/T	T/T	T/T	no data	3	Creb3l1	splice	—
2	161523850	het	C/C	C/G	C/C	C/C	C/C	no data	1	Ptprt	splice	—
4	40933721	het	T/T	T/C	T/T	T/T	T/T	no data	1	Nfx1	splice	—
4	137106002	het	A/A	A/T	A/T	A/A	A/A	no data	3	Hspg2	T→S	0.024
6	113233356	het	G/G	G/A	G/A	G/A	G/A	no data	3	Cpne9	E→K	0.987
9	61783268	het	A/A	A/G	A/A	A/G	A/A	no data	3	Kif23	V→A	0.999
10	78045769	het	G/G	G/T	G/G	G/G	G/G	no data	1	Ilvbl	G→V	0.407
19	39808875	het	T/T	T/C	T/T	T/C	T/T	no data	3	Cyp2c68	I→V	0

In mouse spermatogonial stem cells and the mice conceived from the resulting sperm, ENU has been found to induce a biased set of nucleotide substitutions. Several previous studies have shown an abundance of TA–CG transitions and TA–AT transversions (ranging between 36–43% and 22–44% of changes, respectively [2,12,14,19]) and GC–CG transversions very rarely or never occur [14]. Of the validated 31 true-positive SNVs shown in table 1, 35.5, 38.7 and 0 per cent were TA–CG, TA–AT and GC–CG changes, respectively. Of the remaining 19 non-replicated, false-positive SNV calls, 26.3, 0 and 15.8 per cent were TA–CG, TA–AT and GC–CG changes, respectively.

Exome analysis of the G2 parents of our G3 *nimbus* proband mouse would be expected to reveal all the true ENU variants present in the proband mouse. Likewise, approximately half of the true variants should have also been inherited by the G3 sibling of the proband. Figure 3*c* shows a Venn diagram detailing the overlap between the SNVs called in the exome sequence of the two parents and sibling compared with those in the pooled technical replicate exome sequence of the proband. As expected, all of the seven homozygous mutations called in the proband or its sibling (three in proband + four in G3 sibling) were also called in heterozygous state in both parents. Of the total of 31 validated mutations present in the G3 proband, 28 were called in one or both parents (table 1). Inspection of the sequence data for the two parent G2 exomes revealed that the false-negative mutations were present, but the number of variant reads fell below the required coverage and/or read ratio thresholds used for SNV calling. That three of the 31 true mutations were not identified in one or more of the replicate analyses indicates a technical false-negative rate of 9.7 per cent per exome analysis. However, this estimate does not accommodate the percentage of true mutations that might be missed consistently because they lie in exons that are inefficiently captured and exhibit low sequence coverage.

The false-negative rate of SNV detection can also be estimated from the distribution of sequence read depths generated at random across a whole genome. The depth of reads obtained from random short-read sequencing approximates a Poisson distribution and the probability of observing both alleles at a single site in a diploid genome is a binomial function of read depth [20,21]. A combination of these two distributions can be used to estimate the false-negative SNV call rate based simply on the mean read depth [20–22]. While the distribution of read depths obtained from exome capture appears to be mostly Poisson distributed, this approximation does not hold for sites that are poorly enriched by hybridization to exomic baits (see electronic supplementary material, figure S2), which are also the sites where low coverage is likely to result in the greatest incidence of false-negative calls. In order to estimate an accurate false-negative SNV call rate we used the observed distribution of sequence read depths rather than that derived from a Poisson function. In this manner, we calculated the false-negative SNV call rate in the *nimbus* G3 proband mouse as 21.3 per cent. The average read depth from this dataset was 39.5, but 14.6 per cent of CCDS exomic bases were not covered at all, this being the major source of missing SNV calls. Increasing the amount of sequence data does reduce the false-negative rate slightly, but still a large number of genomic sites will remain poorly covered, either owing to it being difficult to design capture baits to these regions or owing to extreme GC content reducing the efficiency of hybridization of some areas of the genome (data not shown).

**Figure 4.**
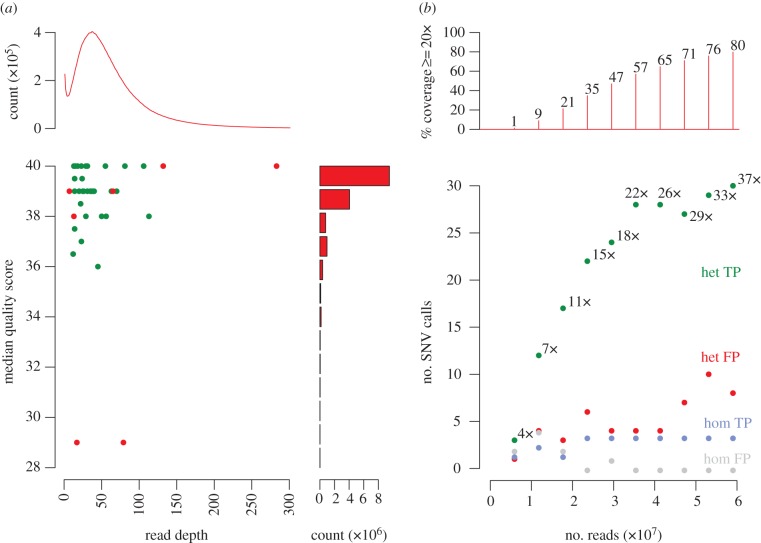
The influence of sequence quality scores and read depth on the identification of true-positive and false-positive SNVs. (*a*) False-positive calls with respect to read depth and quality score, shown for a single exome dataset generated from the G3 *nimbus* mouse (technical replicate 1 from figure 3). Variant calls on this dataset were compared with the PCR-validated true-positive and false-positive SNVs called in the technical replicate exome datasets of the G3 *nimbus* proband. Green and red points are true- and false-positive SNV calls, respectively. The distribution of read depth frequencies over all exonic bases is indicated by the red line in the top graph. The red bars in the right-hand graph indicate the distribution of quality scores also ascertained for all exonic bases. (*b*) Results of simulation experiment performed to generate random subsets of a single exome dataset, being one of the triplicate exome runs for the *nimbus* proband (technical replicate 1). The panel shows tallies of true-positive heterozygous (green), false-positive heterozygous (red), true-positive homozygous (blue) and false-positive homozygous (grey) SNV calls plotted against the number of input reads, which are incremental proportions of an Illumina GAIIx lane. Numbers alongside the green dots indicate the median read depth determined for each true-positive data point. Plotted above are the proportions of the exome covered at 20× depth or better for each proportion of the input read set.

Taking the SNV calls from a single replicate exome from the *nimbus* proband G3 mouse, we investigated whether or not validated true- and false-positive SNVs differed in sequence coverage or quality. Figure 4*a* shows that false-positive SNVs had unusually high or low read depth, or had lower quality scores, relative to the depth and quality of reads across all exonic nucleotides. However, in these data the read depths and quality scores of false-positive variants overlap with those of true-positive calls. While we have chosen to minimize the false-negative rate as much as possible, if it were desirable to reduce the false-positive call rate at the expense of the false-negative rate, this could potentially be achieved with more stringent filtering against read depth and quality score.

To evaluate how deeply an exome should be sequenced, we simulated an exome sequencing experiment where incremental proportions of one lane of exome sequence reads were randomly sampled from a full lane of G3 *nimbus* exome data (figure 4*b*). While reliable homozygous variant calls (blue dots in figure 4*b*) were made at even shallow read depths, a substantially greater depth was required for reliable heterozygous variant calls. True-positive heterozygous variant calls (green dots in figure 4*b*) increased significantly with increasing depth up to a total of 30 million reads. Ninety-three per cent of true-positive mutations were detected with 35–40 million reads (22–26 times median depth). With increasing the read depth beyond this value, relatively few additional true positives were called but the number of false-positive heterozygous SNV calls doubled.

### 3.3. Functional validation of causative mutation in *nimbus* strain

**Figure 5.**
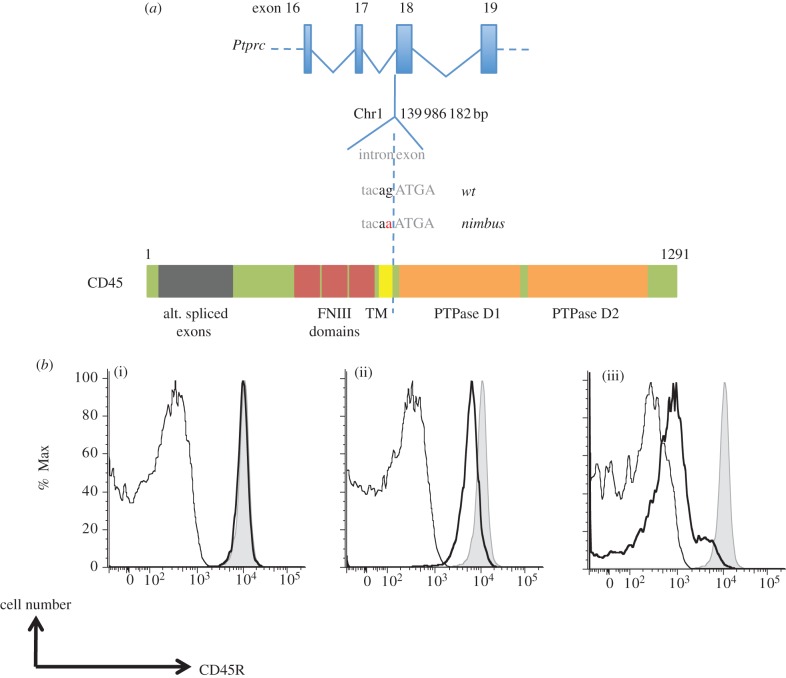
*Nimbus* results from a loss of function mutation in the *Ptprc* gene. (*a*) Schematic diagram showing the location of single nucleotide mutation at Chr1:139986183 at the +1 intronic position of the exon 17 splice donor sequence and the location of the corresponding region in the encoded CD45 protein (TM, transmembrane domain; FNIII, fibronectin III-like domain; PTP, protein tyrosine phosphatase). (*b*) Loss of CD45 protein expression. Bold black lines show flow cytometric staining with antibody to the B-cell-specific CD45R isoform on IgM^+^, IgD^+^ B lymphocytes in blood from (i) *Ptprc^+/+^* wild-type (wt), (ii) *Ptprc^nimbus/+^* heterozygous or (iii) *Ptprc^nimbus/nimbus^* homozygous mouse, compared with negative control staining on CD3+ T cells in the same mouse (thin black line) and compared with positive control staining with the same antibody on B cells in a wt mouse (grey shaded area).

To identify the mutation causing the recessive lymphopaenia phenotype in the *nimbus* strain, we performed Amplifluor assays on each of the three homozygous mutations identified in the proband exome sequence to trace their inheritance in the pedigree. Homozygosity for a C-to-T mutation identified at Chr1 : 139 986 182 bp was found to co-segregate with the lymphopaenia phenotype (table 2). This change lies 1 bp upstream of exon 18 of the *Ptprc* gene and disrupts the intronic-1 G nucleotide of the consensus splice acceptor sequence [34], which is otherwise absolutely conserved across vertebrates. PCR amplification of the mutant *Ptprc^nim^* mRNA showed the first 14 bp of exon 18 were deleted compared with the spliced wild-type mRNA and putatively the AG nucleotides at +13 to 14 of exon 18 from an alternative splice acceptor site. This altered splicing leads to a frameshift in the mutant transcript from the truncated start of exon 18 onwards. *Ptprc* encodes the CD45 protein, which is a tyrosine phosphatase receptor type C. CD45 is an abundant protein in the plasma membrane of leukocytes and plays critical roles in lymphocyte development in mice and humans (reviewed in [35]). Mice homozygous for the *Ptprc^nim^* mutation indeed had almost no CD45 protein on the surface of their B-lymphocytes (2% of wild-type controls) as measured by flow cytometric staining with antibodies to CD45 (figure 5*b*), while heterozygous mice showed an approximately 50 per cent reduction in the expression of CD45. The lymphopaenia in *nimbus* homozygotes matches that in mice and humans with other null or severe loss-of-function mutations in *Ptprc* [29,36,37].

**Table 2.** Mutations identified using exome sequence data.

sample identifier	capture	hom calls	het calls	causal or incidental mutation	gene	detected zygosity	observed phenotype	published allele	published allele phenotype	ref	chr	coord	ref allele	var allele	AA change	PolyPhen score	observed genotype–phenotype correlation
ENU16CH51a	Agilent	3	46	causal	Prkdc	hom	few T and B cells in blood	Prkdc^SCID^	few B or T cells	[5]	16	15810811	T	A	Y3442→X	—	6 hom affected, 19 het unaffected, 3 wt unaffected
ENU14CH36b	Agilent	14	21	causal	CD22	hom	fewer mature and more immature B cells in blood	Cd22^tm1Eac^	fewer mature B cells	[23]	7	31655399	A	T	C512→X	—	1 hom affected, 2 het unaffected, 1 wt unaffected
ENU16NI19a	Agilent	6	32	causal	Dock2	hom	decreased naive T cells and B cells in blood	Dock2^tm1Tsas^	decreased naive T and B cells in blood	[24]	11	34414481	C	A	E775→X	—	14 hom affected, 16 het unaffected, 5 wt unaffected
ENU16CH85a	NG	2	20	causal	Reln	hom	ataxia and small body size	Reln^rl−tg^	tremors, dystonia and ataxia	[25]	5	21408594	A	G	splice	—	4 hom affected, 2 het unaffected, 6 wt unaffected
ENU16CH17a	Agilent	3	45	causal	Lyn	hom	decreased blood B cells, increased percentage immature	Lyn^Mld4^	decreased B cells	[26]	4	3710143	A	G	T410→A	0.990	10 hom affected, 6 het unaffected, 1 wt unaffected
ENU14CH48	NG	7	62	causal	Prkdc	hom	few T and B cells in blood	Prkdc^SCID^	few B or T cells	[27]	16	15714375	T	C	splice	—	4 hom affected, 12 het unaffected, 3 wt unaffected
ENU16NI24a	NG	9	37	causal	Lepr	hom	obese	Lepr^db^	obese	[28]	4	101452668	T	A	N429→K	1.000	3 hom affected, 17 het unaffected, 24 wt unaffected
*nimbus*	Agilent	3	40	causal	Ptprc	hom	decreased naive T cells and B cells in blood	Ptprc^tm1Holm^	decreased naïve T cells and B cells	[29]	1	139986182	C	T	splice	—	12 hom affected, 18 het unaffected, 3 wt unaffected
ENU16CH71a	NG	10	30	causal	Pax5	hom	few blood B cells	Pax5^tm1Mbu^	arrest of B cell development	[30]	4	44704884	G	A	I78→N	0.266	24 hom affected, 23 het unaffected
ENU18CH65a	NG	4	40	causal	Fcer2a	het	decreased Fcer2a (CD23) on B cells				8	3690110	G	T	C18→X	—	11 hom affected, 29 het intermediate, 20 wt unaffected
ENU16NI3b	NG	7	41	causal	KLRG1	hom	low KLRG1 on NK cells				6	122232913	G	A	5′ UTR	—	20 hom affected, 1 het affected, 9 het unaffected
	"	"	"	incidental	Rab27a	het	coat colour (Ashen)	Rab27a^ash^	grey coat colour	[31]	9	72930272	T	A	W73→R	1.000	3 hom affected,13 het unaffected, 6 wt unaffected
	"	"	"	incidental	Tg	hom	small body size	Tg^cog^	hypothyroidism, goiter, impaired growth	[32]	15	66536802	C	T	R1471→X	—	4 hom affected, 10 het unaffected, 2 wt unaffected
ENU15CH72a	NG	0	13	causal	Ptpn6	het	decreased IgM on mature B cells	Ptpn6^mev^	decreased IgM on mature B cells in hets	[33]	6	124672073	G	A	T464→I	1.000	4 hom affected, 29 het affected, 21 wt unaffected

### 3.4. Identification of causal mutations in 11 additional strains

The successful use of exome analysis to identify causative mutations without meiotic mapping was repeated for 11 other ENU pedigrees with immune disorders or obesity, applying the same analysis to individual exome sequences from proband G3, G4 or G5 mice (table 2). In each of these pedigrees, the causative mutation was revealed solely using exome sequence data followed by SNV-specific Amplifluor PCR typing to correlate the SNV genotypes with the phenotype in the pedigree, without the need for meiotic mapping. The mutations found in each of these strains variously included premature stop codons, disrupted splice donor or acceptor sites and missense changes. The correlation between genotype and phenotype, together with the similar phenotype of independent mutant alleles of the same genes, provided strong corroboration that the mutations identified by exome sequencing were indeed responsible for the phenotypes observed in these mice.

A mean of 6 homozygous and 36 heterozygous mutations were called in the exome sequence of each of the proband individuals from the strains analysed in table 2. These numbers are small enough to contemplate exhaustive validation of each SNV and typing of siblings by Amplifluor PCR assays to test phenotype–genotype concordance, although in many cases a knowledge of the function of the mutant genes allowed candidate mutations to be prioritized. Of the nine strains for which a recessive mutant was sought, the causative variant needed to be selected from on average only 6.4 (*σ* = 3.8) candidate mutations. Two of the strains required the causative variant to be identified in a heterozygous form. In these two strains the heterozygous candidate mutation lists were tractably just 40 and 13 variants long.

The incidental mutations revealed by exome sequencing of proband mice in each pedigree represent a remarkable resource for gene-driven testing for other phenotypes. On average, 35.5 (*σ* = 13.7) heterozygous exonic mutations were identified in the G3, G4 and G5 mice presented in table 2. Applying the false-positive rate of 19.4 per cent deduced above, on average each G3, G4 or G5 mutant mouse will carry around 29 incidental heterozygous mutations. This gene-driven strategy was successfully reduced to practice in the strain ENU16NI3b, where the original phenotype of low KLRG1 protein on the surface of NK cells occasionally co-occurred with ashen coat colour or stunted growth, neither of which could be explained by a mutation in the KLRG1 gene. With reference to the mutation list obtained from exome sequencing of the G3 proband mouse in this strain, two additional incidental mutations were found by Amplifluor PCR to segregate with each incidental phenotype. A homozygous missense mutation in *Rab27a* co-segregated with ashen coat colour in this pedigree, and an independent *Rab27a* mutation has previously been shown to cause the same trait through a defect in melanosome transport [31]. A homozygous nonsense mutation in the thyroglobulin gene, *Tg^R1471X^*, was found to co-segregate in the same pedigree with stunted growth, and this complements an independent study that showed that a spontaneous missense mutation in the *Tg* gene caused stunted growth, hypothyroidism and goiter in an AKR mouse substrain [32]. The new *Tg^R1471X^* strain provides a C57BL/6j mouse model for human thyroid dyshormonogenesis 3 syndrome (OMIM: 274700), which was first shown to result from a similar R1510X nonsense mutation in thyroglobulin [38].

### 3.5. Mutant first-generation mouse resource

The sensitivity and specificity of detecting heterozygous de novo mutations established above opened up a broader strategy to develop mouse experimental models based on tracking specific  mutations in gene-driven phenotypic screens, as had been done for the *Tg* and *Rab27a* mutations. To make it possible to do this in a systematic way, we extended the exome sequencing approach to identify novel protein-changing mutations arising in the G1 founders of ENU mutagenized pedigrees, prior to any phenotypic screening or selection of their G2 and G3 progeny, and when all the mutations are heterozygous (figure 1). We sequenced the enriched exomes of eight different G1 mice as a bar-coded, pooled sample on an Illumina HiSeq sequencing run. This provided a greater number of reads per exome than the datasets generated on the GAIIx sequencers, and yielded better than 20 times sequence depth over 80.7 per cent (*σ* = 1.8%) CCDS exons. As expected, very few homozygous variants were identified in the filtered variant lists, presumably being rare variants previously unobserved in the parental C57BL/6j stock. The numbers of heterozygous variants in the G1 mice (*μ* = 59.6, *σ* = 13.1) were higher than those found in G3, G4 or G5 mice (*μ* = 36.5, *σ* = 13.7; table 2), which was as expected since a fraction of ENU-induced alleles will be lost in each subsequent generation owing to random drift and purifying selection. Hence, given the information presented in figure 4*b*, we would expect that the majority of true ENU-induced mutations have been detected from these datasets.

**Figure 6.**
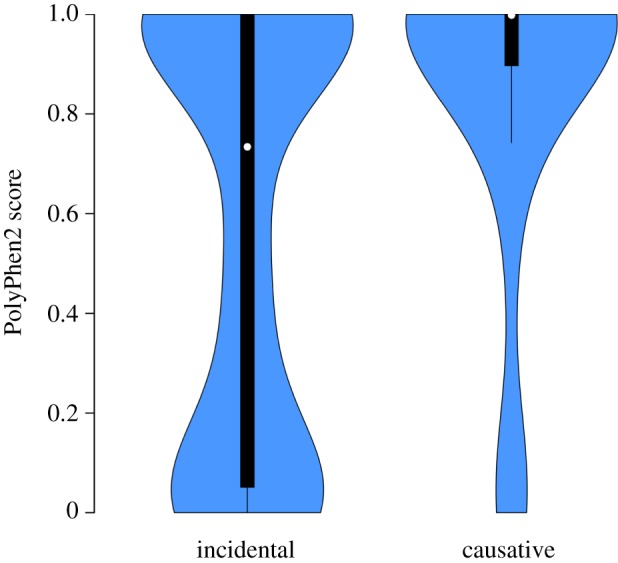
Violin plot comparing PolyPhen2 scores for incidental and causative mutations. The black bars represent a boxplot where 50% of values lie within the main bar. The white dot indicates the median polyphen value for each set of scores. The blue region is a kernel density plot representing the distribution of PolyPhen2 scores. The numbers of mutations included in the plot were: incidental mutations, *n* = 325 and causative mutations, *n* = 40. A Mann–Whitney test for the equality of the mean PolyPhen2 score of the incidental and causative mutations indicated a significant difference in score (*W* = 4168, *p* = 0.0000862).

Of the 454 unique mutations detected across these eight G1 mice, 18 (4%) created a premature stop codon, 65 (14%) putatively disrupted an mRNA splice donor/acceptor site and 370 (81%) caused an amino acid substitution (see electronic supplementary material, table S4). We altered PolyPhen2 [39] to use mouse sequence databases (rather than the default human inputs) and calculated scores for missense G1 mutations. Figure 6 shows a comparison of these scores with those calculated for a set of previously characterized ENU-induced mutations known to cause immunological traits. For the causal missense mutations, PolyPhen2 correctly assigned a very high score (greater than 0.95) of ‘probably damaging’ to 75 per cent and an intermediate to high score (0.44–0.95) of ‘possibly damaging’ to a further 15 per cent. This result validates the predictive accuracy of PolyPhen2 when applied to novel mouse mutations. Of the 370 de novo missense mutations identified in G1 mice, 134 (36%) were assigned a ‘probably damaging’ score of greater than 0.95 and 59 (16%) were classified as ‘possibly damaging’ with a score of 0.505–0.897. The genes affected by these 272 potentially damaging mutations include those known to cause human disease through to entirely unexplored genes with intriguing expression patterns and protein domains (see electronic supplementary material, table S3). By identifying de novo ENU mutations in G1 founders in this way and then breeding, genotyping and phenotyping their G2 and G3 offspring, this approach provides an immediate source for new experimental models for understanding human diseases and traits.

## 4. Discussion

The pursuit of gene function that starts with the identification of medically important phenotypes displayed by individual mammals (the so-called forward-genetics) has until now been constrained by the time-consuming and expensive bottleneck of mapping these traits to their underlying genetic cause. Conversely, reverse genetics approaches based on knocking out individual genes in embryonic stem cells remain constrained by a comparably time-consuming and expensive bottleneck of converting the embryonic stem cells into a pedigree of mice that can be phenotypically evaluated. Here we have shown that exome capture followed by massively parallel DNA sequence analysis reliably identifies the majority of homozygous and heterozygous ENU-induced mutations. Not only does this eliminate the bottleneck to forward genetics by identifying causal mutations without the need for meiotic mapping, but also it bypasses a key restriction for reverse genetics by revealing thousands of possibly damaging mutations in live-breeding C57BL/6j mouse pedigrees that are immediately available for experimental analysis of gene function.

By technical and biological replication of exome analyses and confirmation of individual SNV calls by PCR, we have shown that both homozygous and heterozygous protein-changing mutations induced by ENU de novo in live-breeding pedigrees of C57BL/6j mice can be called reliably with an estimated sensitivity of 78.7 per cent and a specificity of 80.6 per cent. In 11 separate C57BL/6j mutant strains from forward genetics screens for immune system disorders or obesity, we were able to bypass the need for meiotic mapping and identify short lists of protein-changing ENU-induced mutations that were heterozygous or homozygous in proband individuals from these pedigrees, among which we were able to identify a causative mutation that explained the immunological or obesity phenotype. In identifying ENU-induced mutations, we found massively parallel sequencing data to be highly reliable and sources of error were predictable, such that by filtering commonly called variants (along with previously observed genetic variation) we were able to restrict the false-positive call rate to less than 20 per cent while not incurring a disproportionate false-negative call rate. In terms of the read depth required to reliably identify heterozygous mutations, we found that around 35 million paired-end sequence reads are sufficient to identify more than 90 per cent of these changes.

Fairfield *et al.* [9] have also produced an extensive demonstration of exome capture and sequencing in mice to identify causative mutations. In their study, exome sequence data were used in combination with meiotic mapping information to identify causal mutations without a large validation burden. Our results both confirm and extend this study. Laudably, the Fairfield *et al.* [9] study describes three mutant strains where they did not identify a causal mutation, even with the aid of meiotic mapping information. They speculated that, in those strains where the causal mutation could not be identified, it probably lay outside the chromosomal regions enriched by exome capture. Our analysis provides further insight into this problem and shows that, in approximately one of five mouse strains, we can expect a causal mutation to remain undetected owing to it not being efficiently captured prior to sequencing and/or subsequently detected. We found that solution capture methods commercialized by Agilent and Nimblegen are both effective at specifically concentrating the coding part of the mouse genome, but that a consistent approximately 15 per cent portion of exonic regions is absent from subsequently sequenced reads, regardless of how deeply the captured DNA is sequenced. This may be a fundamental limitation of exome enrichment technologies, perhaps indicating that some genomic regions may be resistant to efficient hybridization with capture baits and/or the PCR amplification steps in the capture and library preparation protocols. From analysis of exome datasets from related mice, in a small number of cases known heterozygous variants were only poorly detected owing to a very few reads supporting the mutant genotype. This effect may indicate that in some local sequence contexts the mutant genotype is out-competed by the reference genotype during sequence capture.

Mutated C57BL/6j inbred mice provide an ideal system for tackling the challenges of identifying rare, de novo mutations from a background of normal genetic variation. While the laboratory mouse is an inbred organism with very little genetic variation, we found that it was necessary to control for even this small amount of variation through a series of data filtering strategies employing catalogues of known strain variants and other sources or recurring false positives in order to identify true mutations with high specificity. Given sufficient data for a specific mouse strain (10–20 individual exome sequences), this strategy of cataloguing recurrent variants has also proven effective in identifying ENU-induced mutations in mice out-crossed to strains other than C57BL/6j (data not shown). We found that detection of ENU-induced mutations can be further enhanced by technical replication of exome analysis and by biological replication taking advantage of heritability information in closely related individuals. Taken together, this information makes pathogenic mutation detection in outbreeding mammals (such as humans) a more tractable possibility.

We have shown that it is feasible to also perform these exome analyses in multiplexed, bar-coded samples from many separate G1 founder mice. This makes it straightforward to analyse the exomes of hundreds of G1 founder mice per year and propagate the mutations they carry in live-breeding pedigree structures such as the ones employed here (figure 1). Given the number of protein-changing mutations we identified in each G1 mouse (table 3), a live-breeding resource of 350 pedigrees bred for two generations from 700 G1 mice each year would reveal 42 000 new protein-changing mutations per year, of which around half are expected to be deleterious. Hence, reliable identification of induced mutations has the potential to transform genetic screens of genes of unknown function and produce mouse models of hundreds of human diseases.

**Table 3.** Sequencing statistics and variant calls for G1 mice.

sample identifier	total reads sequenced	CCDS on-target efficiency	median read depth over CCDS exons	CCDS bases covered 5 times or better depth (%)	CCDS bases covered 20 times or better depth (%)	raw variant calls	filtered homozygous variant calls	filtered heterozygous variant calls
MMP-1	94013861	0.539	87	85.9	82.3	10 463	3	55
MMP-2	96872136	0.532	89	86.0	82.6	11 834	0	59
MMP-3	97528301	0.538	89	85.9	82.3	11 396	2	79
MMP-4	71404847	0.536	66	85.3	79.7	9349	0	54
MMP-5	72606249	0.525	64	84.9	78.2	13 404	1	74
MMP-6	92123751	0.531	84	85.9	82.1	10 328	0	42
MMP-7	71220780	0.531	65	85.3	79.5	9050	3	68
MMP-8	67858625	0.537	62	85.2	79.0	8929	0	46

## 5. Material and methods

### 5.1. Mutant mouse generation

The *nimbus* mouse strain was generated by treating pure C57BL/6j male mice with the mutagen ENU at the Australian Phenomics Facility of the Australian National University as previously described [10]. Briefly, adult male animals received 90 mg of ENU per kilogram of body weight by three weekly intraperitoneal injections. Once fertility was regained after a further eight weeks, the animals were mated with C57BL/6j females to generate G1 offspring carrying a unique cohort of heterozygous SNVs. A subset of SNVs was brought to homozygosity through unrelated G1 crosses followed by intercrossing to G3 (as shown in figure 1). A peripheral blood screen for lymphocyte subsets identified the *nimbus* strain at G3 as displaying a mild lymphopaenia. All other mutated mouse strains sequenced were generated via this protocol.

### 5.2. Exome enrichment and sequencing

DNA was extracted from ear tissue of affected mice and 3.5 μg prepared as paired-end genomic libraries (PE-102-1001: Illumina, San Diego, CA). Technical replicates were produced from the same DNA sample. Exome enrichment was performed using either the SureSelect Mouse Exome kit (G7550A-001: Agilent, CA) or the SeqCap Mouse Exome kit (early access: Nimblegen, Madison, WI) following the manufacturer protocols. Four amplification cycles were used in the library pre-capture PCR using Herculase II fusion polymerase (600677, Stratagene) and eight cycles in the post-enrichment amplification for both capture technologies. Enriched libraries were diluted to 10 nM concentrations before further dilution to 7–8 pM for cluster generation and sequencing-by-synthesis on either the Illumina Genome Analyser IIx as 75 bp PE reads or the Illumina HiSeq as 100 bp reads. Each library sequenced on an Illumina GAIIx was sequenced on a single lane of an eight-lane flow-cell, whereas libraries sequenced on the Illumina HiSeq were multiplexed in a pool of 10 samples and sequenced together, and disambiguated using sample bar-coding.

### 5.3. Single-nucleotide variant detection workflow

A custom workflow was developed to process sequence reads to detect ENU-induced mutations. This workflow holds together a number of open-source analysis tools and employs a Perl code-base to perform custom filtering, reporting and job process control (figure 2*a*). BWA (v. 0.5.9-rc16; [16]) with default settings was chosen to align paired-end reads to the reference mouse genome (mm9/NCBIM37). Reads aligning to multiple genomic locations were removed and raw SNV calls were made using SAMTools (v. 0.1.15; [17]) with parameters set to allow a less conservative calling rate than the default settings, which significantly involved disabling the base alignment quality filtering function. Raw SNV calls were classified as homozygous or heterozygous on the basis of the ratio of alleles (hom > 0.8 variant allele; het two alleles > 0.3) and then annotated as to whether they were also present in dbSNP (v. 128; http://www.ncbi.nlm.nih.gov/snp/), whether they commonly occurred in our exome data and, where appropriate, whether they were strain-specific variants identified from the Sanger Institute mouse genomes sequencing project (http://www.sanger.ac.uk/resources/mouse/genomes/). Commonly occurring variants were collated from all exome data collected by our laboratory. Further annotation of variants was performed to determine overlap with CCDS exons [18] and denote non-synonymous changes (using Annovar [40]). Changes that lay in potential splice donor–acceptor sites immediately adjacent to exon boundaries (out to 10 intronic bases) were also annotated. Using these annotations, we filtered the variant list to only include non-synonymous or splice donor–acceptor site changes that were novel to a particular sample. From this filtered list of variants, for each exome a list of genes containing more than one variant was compiled for each sample and then used to further filter variants across all samples that were found in these multi-SNV genes.

### 5.4. Variant validation

SNVs were validated using Amplifluor assays (Chemicon, Temecula, CA). Primers were designed using the Assay architect online tool (http://apps.serologicals.com/AAA/mainmenu.aspx). Fluorescent intensities were detected using a Fluostar optima (BMG). The individual affected mice used in the study and a C57BL/6j control were analysed for each SNV assay.

## Supplementary Material

Supplemental Table S1

## Supplementary Material

Supplemental Table S2

## Supplementary Material

Supplemental Table S3

## Supplementary Material

Supplemental Figure S1

## Supplementary Material

Supplemental Figure S2
