# Supplementary material for: Massively parallel sequencing of the mouse exome to accurately identify rare, induced mutations: an immediate source for thousands of new mouse models
Source: Open Biol. 2012 May;2(5):120061. doi: 10.1098/rsob.120061 (PMC3376740; doi:10.1098/rsob.120061)
Supplement: Supplemental Figure S1 [file rsob120061-s4.doc]

**
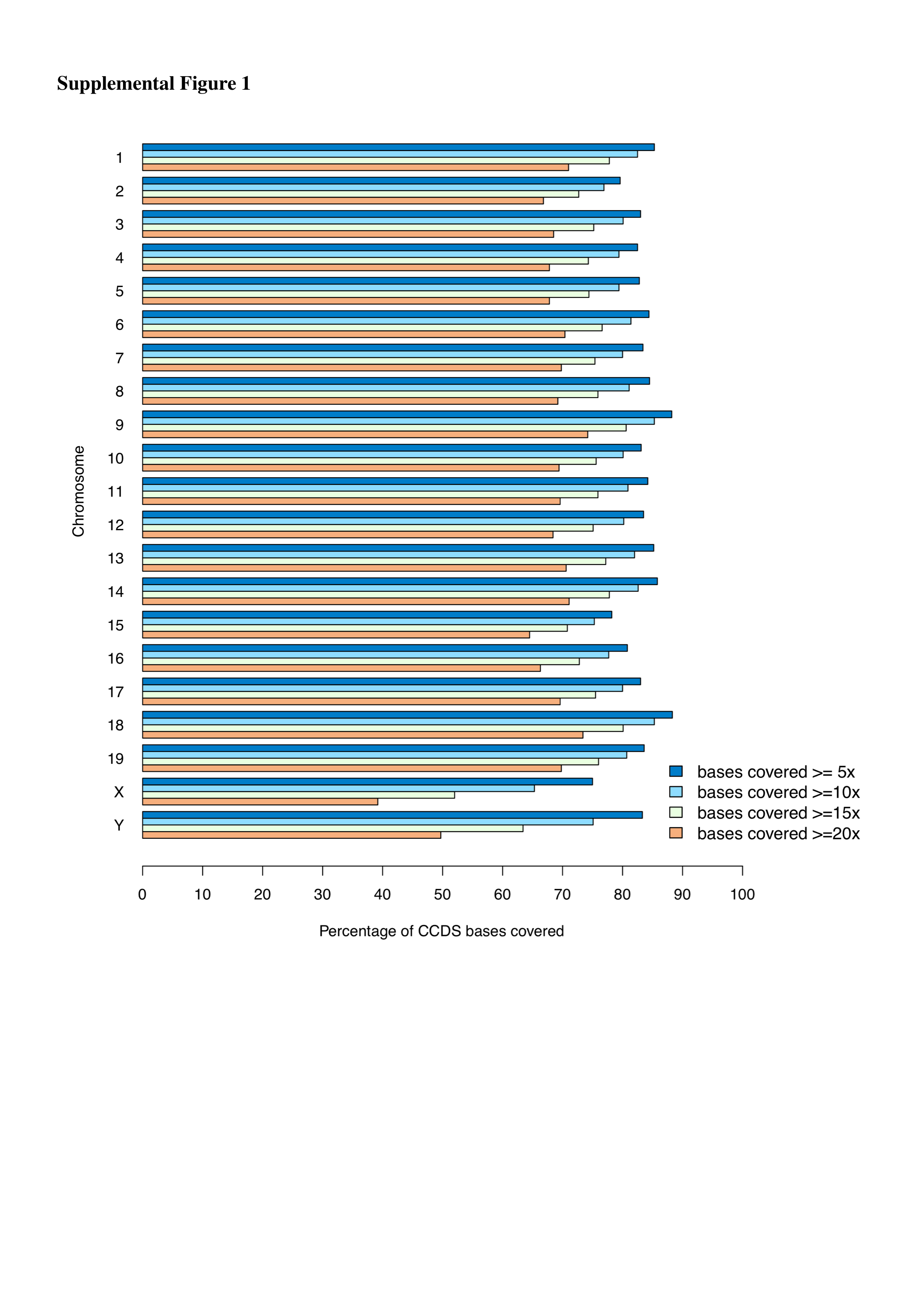
**

**Supplemental Figure S1.** Percentages of covered nucleotide sites in CCDS exons presented by chromosome at increasing read depth cutoffs. Amber, yellow, light blue and dark blue bars show percentage of CCDS bases covered by 20, 15, 10 or five reads or more, respectively. These coverage values are taken from a dataset of 24 million read pairs sequenced from DNA enriched 30-fold by exome capture (12 million on-target read pairs).
