# Supplementary material for: Massively parallel sequencing of the mouse exome to accurately identify rare, induced mutations: an immediate source for thousands of new mouse models
Source: Open Biol. 2012 May;2(5):120061. doi: 10.1098/rsob.120061 (PMC3376740; doi:10.1098/rsob.120061)
Supplement: Supplemental Figure S2 [file rsob120061-s5.doc]

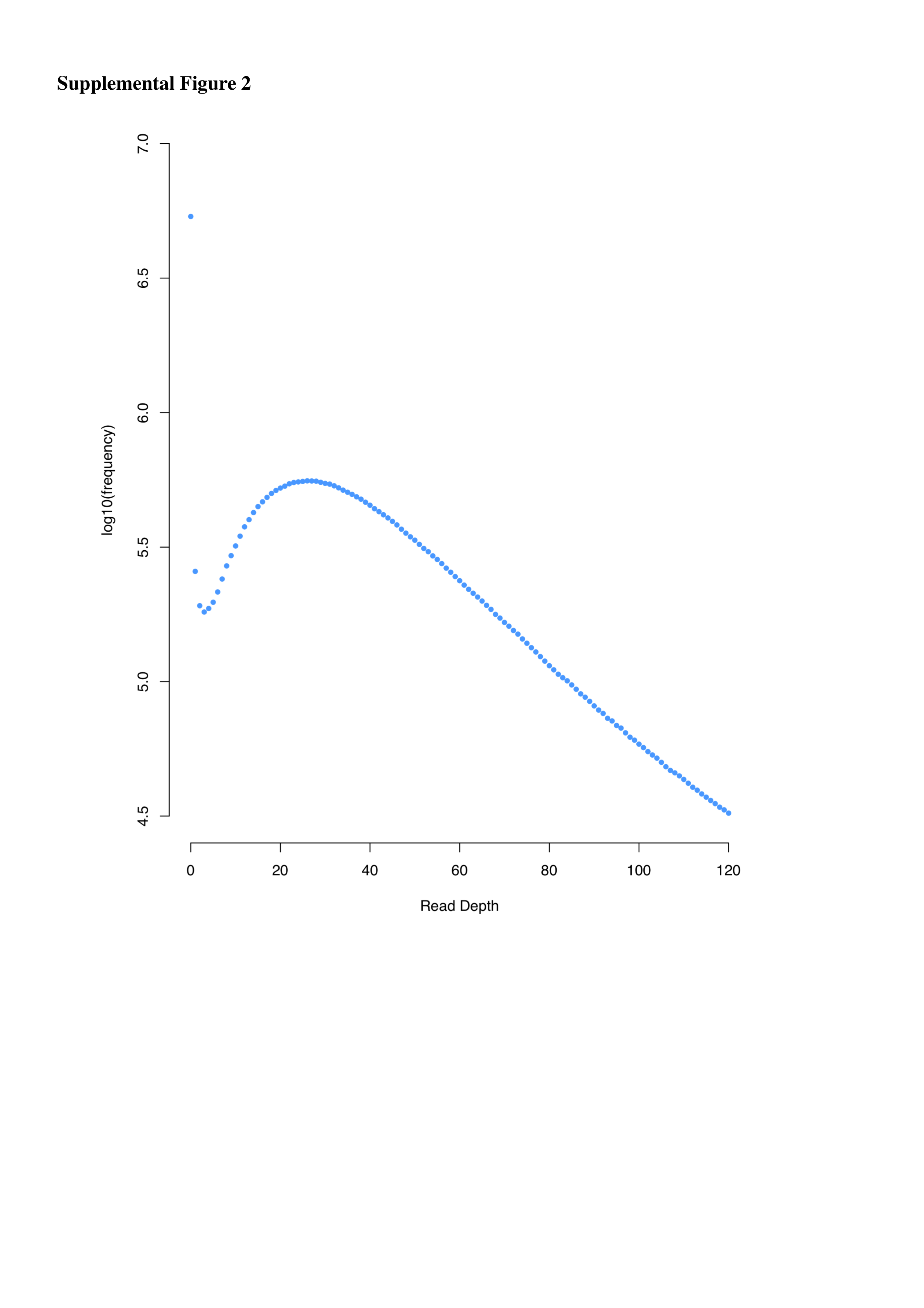


**Supplemental Figure S2.** Distribution of sequence read depths observed across each coding base in the genome (annotated CCDS exons) from a dataset totalling 24 million read pairs, enriched 30-fold for exonic DNA.
